# Impact of CMV Infection on Natural Killer Cell Clonal Repertoire in CMV-Naïve Rhesus Macaques

**DOI:** 10.3389/fimmu.2019.02381

**Published:** 2019-10-09

**Authors:** Lauren L. Truitt, Di Yang, Diego A. Espinoza, Xing Fan, Daniel R. Ram, Matilda J. Moström, Dollnovan Tran, Lesli M. Sprehe, R. Keith Reeves, Robert E. Donahue, Amitinder Kaur, Cynthia E. Dunbar, Chuanfeng Wu

**Affiliations:** ^1^Translational Stem Cell Biology Branch, National Heart, Lung, and Blood Institute, National Institutes of Health, Bethesda, MD, United States; ^2^Institute of Hematology, Tongji Medical College, Union Hospital, Huazhong University of Science and Technology, Wuhan, China; ^3^Perelman School of Medicine, University of Pennsylvania, Philadelphia, PA, United States; ^4^Center for Virology and Vaccine Research, Beth Israel Deaconess Medical Center, Harvard Medical School, Boston, MA, United States; ^5^Tulane National Primate Research Center, Covington, LA, United States; ^6^Ragon Institute of Massachusetts General Hospital, MIT, and Harvard, Cambridge, MA, United States

**Keywords:** NK cells, cytomegalovirus, adaptive memory, barcoding, clonality

## Abstract

Recent functional, gene expression, and epigenetic studies have suggested the presence of a subset of mature natural killer (NK) cells responsible for maintaining NK cell memory. The lack of endogenous clonal markers in NK cells impedes understanding the genesis of these cell populations. In humans, primates, and mice, this phenotype and memory or adaptive functions have been strongly linked to cytomegalovirus or related herpes virus infections. We have used transplantation of lentivirally-barcoded autologous hematopoietic stem and progenitor cells (HSPC) to track clonal hematopoiesis in rhesus macaques and previously reported striking oligoclonal expansions of NK-biased barcoded clones within the CD56^−^CD16^+^ NK cell subpopulation, clonally distinct from ongoing output of myeloid, B cell, T cell, and CD56^+^16^−^ NK cells from HSPC. These CD56^−^CD16^+^ NK cell clones segregate by expression of specific KIR surface receptors, suggesting clonal expansion in reaction to specific environmental stimuli. We have now used this model to investigate the impact of rhesus CMV(RhCMV) infection on NK clonal dynamics. Following transplantation, RhCMV^neg^ rhesus macaques display less dominant and oligoclonal CD16^+^ NK cells biased clones compared to RhCMV^pos^ animals, however these populations of cells are still clearly present. Upon RhCMV infection, CD16^+^ NK cells proliferate, followed by appearance of new groups of expanded NK clones and disappearance of clones present prior to RhCMV infection. A second superinfection with RhCMV resulted in rapid viral clearance without major change in the mature NK cell clonal landscape. Our findings suggest that RhCMV is not the sole driver of clonal expansion and peripheral maintenance of mature NK cells; however, infection of macaques with this herpesvirus does result in selective expansion and persistence of specific NK cell clones, providing further information relevant to adaptive NK cells and the development of NK cell therapies.

## Introduction

Natural killer (NK) cells are classically-defined as circulating and tissue-resident immune effectors responsible for production of regulatory and supportive cytokines as well as the killing of infected and malignant cells. NK cells have been historically considered innate effector cells, lacking both the rearranged diverse antigen receptors present in B and T cells conferring specificity and the self-renewal and/or longevity of reactive clones necessary to confer immune memory. However, there is increasing direct and correlative evidence for properties of NK cells providing adaptive memory in mice, non-human primates, and humans in response to viral infection, immunization, or cytokine stimulations (1–8). Functional, gene expression, and epigenetic studies have defined subsets of natural killer cells potentially responsible for these adaptive properties (1, 6, 9–11).

Clonal expansions are integral in understanding T and B cell memory; yet, the lack of endogenous clonal markers in NK cells has impeded understanding of the genesis and maintenance of putative memory or adaptive NK cell populations. Efforts to elucidate mechanisms underlying NK memory have focused on analyzing expression patterns and “pseudo-clonal” expansions of NK cells with specific patterns of surface receptors known to interact with MHC molecules or viral targets, specifically Ly49 receptors in mice (9) or killer immunoglobulin-like receptor (KIR) in humans. The pathways resulting in heterogeneity of NK cell functions are complex and not completely understood, with the character of responses to the environment appearing to depend on the timing and amalgamation of expression of activating and inhibitory cell surface receptors.

Mouse NK cells are phenotypically and functionally distinct from human NK cells, limiting extrapolation from this model organism. The Ly49 family of receptors in the mouse has been shown to have some analogous functions to human KIRs; however in terms of their structure, these molecules are highly dissimilar (12). In contrast to murine models, non-human primates, specifically rhesus macaques (RMs), are phylogenetically closely related to humans, and their NK cells share many phenotypic and functional properties with human NK cells (13–15).

We have recently utilized genetic barcoding of transplanted autologous RM hematopoietic stem and progenitor cells (HSPCs) to track hematopoiesis at a clonal level *in vivo* (16, 17). Previously, we observed striking expansions of circulating mature CD56^−^CD16^+^ NK cell clones, clonally distinct from myeloid, B cell, T cell, and CD56^+^16^−^ NK cells implying an independent differentiation and maintenance pathway distinct from ongoing production from HSPC, perhaps due to peripheral self-renewal (18). Groups of peripheral expanded clones appeared rapidly following transplantation and showed variable degrees of waxing and waning over time, as if in response to environmental stimuli, similarly to peripheral mature effector T cell clonal dynamics. Strikingly, these expanded NK clones segregated by KIR expression long-term, with specific clones either expressing or not expressing specific KIRs, for the first-time linking expression of specific interacting receptors with clonal expansions and suggesting a potential explanation for maintenance of NK memory. The concept of NK memory was further strengthened by a study showing evidence for antigen-specific NK cell memory following SIV/HIV vaccination in RM indicating the existence of functional memory NK cells (19).

In humans, recent studies have demonstrated populations of mature adaptive NK cells with a distinctive signaling, functional, and transcription factor profiles along with epigenetic characteristics similar to T effector cells that closely correlated with seropositivity for the herpesvirus cytomegalovirus (CMV) (10, 11). Expansions of “pseudoclonal” KIR-segregated NK cells expressing maturation markers such as CD57 and the activating receptor NKG2C have been linked to CMV reactivation post-allogeneic transplantation (20). In the context of reactivation of CMV post-transplant, increases in the NKG2C+ population persisted over time (21, 22). Further, NKG2C gene copy number variation has been shown to play a role in the human NK cell response to CMV infection (23, 24).

Rhesus CMV (RhCMV) has been considered an emerging animal model for studying human CMV due to close phylogenetic relationship, immunogenicity, and identical life cycles, including latency and reactivation following immunosuppression (25). Virtually 100% of RM in the wild or reared in standard captive breeding populations become RhCMV positive by 1 year after birth (26). The RMs previously studied in our barcoded transplantation model were all RhCMV seropositive. We hypothesized that the massive clonal expansions arising post-transplantation may have arisen wholly or in part in response to RhCMV reactivation. We have now used this model to investigate the impact of RhCMV infection on NK cell clonal dynamics and phenotypic subsets by transplanting two RhCMV naïve monkeys with autologous barcoded HSPCs and tracking NK clonal dynamics post-transplantation in comparison to historical barcoded RhCMV^pos^ recipients. To then directly test the relationship between RhCMV infection and NK clonal dynamics, we infected these RhCMV^neg^ animals with RhCMV 9 months post transplantation. Our results provide new insights into NK adaptive features and clonal dynamics related to RhCMV infection and details the phenotype of a model relevant to the human clinic.

## Materials and Methods

### Rhesus Macaque Autologous HSPC Transplantation

Animal studies were carried out on protocols approved by the National Heart, Lung, and Blood Institute Animal Care and Use Committee. Indian-origin RhCMV^neg^ RMs (*n* = 3) were obtained from the expanded specific-pathogen free colony maintained at the Tulane National Primate Research Center and confirmed to be RhCMV-seronegative by whole virion ELISA screening for RhCMV-specific IgG antibodies. These animals were housed in isolation from RhCMV^pos^ RMs and special precautions were taken to maintain their RhCMV^neg^ status before and after transplantation and before RhCMV inoculation, including use of one RhCMV^neg^ animal as a blood donor for the two transplanted RhCMV^neg^ macaques following conditioning radiation and before engraftment.

Peripheral blood CD34+ HSPCs were mobilized, collected via apheresis, immunoselected, and transduced with diverse barcoded lentiviral libraries as described (16–18, 27). Following transduction, CD34+ HSPC were infused into autologous recipients conditioned with 10 Gy total body irradiation.

### RhCMV Infection and Monitoring

RhCMV strain 180.92 (28) was used to infect animals in this study. The virus stock of RhCMV 180.92 used for experimental infection of RhCMV-seronegative rhesus macaques was derived after transfection of virion DNA purified from infected cells into primary rhesus macaque fibroblast lines as previously described (29) (2 × 10^6^ TCID50 was slow thawed on ice, reconstituted with RPMI (Thermo Fisher, cat# 11875119) to a final volume of 1 ml, and given as a slow IV push. Immediately following inoculation, infected animals were housed in a regular specific pathogen free (SPF) room and separated from remaining RhCMV^neg^ animals.

RhCMV DNA copy numbers were determined via real-time qPCR as described. DNA was extracted from 200 μL plasma, urine, or saliva using Qiagen QIAamp DNA Mini kit (Qiagen # 51306) and eluted into 50 μL buffer and three replicates for each sample were amplified using TaqMan Universal PCR MasterMix (Thermo Fisher Cat#4304337). Primers and probes were custom designed for the glycoprotein B gene (UL55) of RhCMV (Table S2). Absolute quantification of RhCMV copy number was calculated based on a standard curve of plasmid containing the target region. RhCMV DNA copy numbers were expressed as copies per ml of plasma or copies per microgram of input DNA in saliva or urine.

RhCMV-specific IgG was measured as previously described (30, 31) by whole virion ELISA, which uses a 96-well plate coated with purified virion preparation of filtered, fibroblast-passaged RhCMV strain 180.92 (32) at 1:3000 dilution. Plasma (1:50 dilution) was incubated in duplicate wells and RhCMV-binding IgG was detected using 1:500 dilution of an HRP-conjugated goat anti-monkey IgG Ab (Santa Cruz Biotechnology, sc-2458) and substrate incubation. The magnitude of the RhCMV specific IgG binding responses is reported as optical density (OD) at 450 nm.

### T Cell Depletion

The recombinant immunotoxin (termed “A-dmDT390-scfbDb(C207)”, referred to as FN18) was produced by fusion of the affinity-matured form of the anti-macaque CD3 monoclonal antibody C207 expressed as a fold-back single chain Fv diabody to a truncated diphtheria toxin (DT390) and produced in yeast (33, 34). This immunotoxin was obtained from the Massachusetts General Hospital-Dana Farber Cancer Center Recombinant Protein Expression and Purification Core Facility, supported by the NIAID/NIH Non-human primate reagent resource program (https://www.nhpreagents.org/NHP/default.aspx). 0.25 ug/kg FN18 was administered via IP push twice daily for 4 days.

### Cell Lineage Purification

Blood samples were processed using Lymphocyte Separation Medium (GE Healthcare, cat# 17144002) to obtain a PB mononuclear cell (PBMC) layer, followed by red blood cell lysis with ACK lysis buffer (Quality Biological, cat# 118156101). PBMCs were stained with a panel of antibodies (Table S1), and specific subsets (Figure S1E) were sorted via fluorescence-activated cell sorting to high purity on a BD FACSAria II instrument. Intracellular staining was performed using the FoxP3/Transcription Factor Staining Buffer (Thermo Fisher Cat#00552300). Subsequent analyses were performed using FlowJo V10 (FlowJo, LLC).

### Clonal Tracking via Barcode Retrieval

Each integrated lentiviral provirus includes a marker copGFP transgene, a 6bp library ID and a 35 or 27 random bp barcode sequence (35). Use of these documented high diversity barcode libraries ensures that each barcode uniquely marks individual engrafting HSPC, as detailed and validated in prior publications (16, 17, 35). By targeting relatively low transduction of HSPC, the majority of HSPC contain a single barcode. The barcode is passed onto each daughter cell and serves as a clonal tag.

DNA was extracted from cell samples using the DNAeasy kit (Qiagen, cat##69506) and 200 ng DNA was amplified via low-cycle PCR with primers bracketing the library ID and barcode (Table S2) with Phusion high fidelity DNA polymerase (Thermo Fisher, Cat #F530L). PCR products were gel purified (Qiagen, cat#28706) and sequenced using the Illumina HiSeq2500 or HiSeq3000 system. Barcode retrieval from the sequencing output was processed to retrieve valid barcodes and analyzed as described, using custom Python and R code which can be accessed at https://github.com/dunbarlabNIH/CMV (16, 17). Only barcodes contributing above a threshold taking into account sequencing errors and sampling constraints were included in analyses (17).

### RNA-Flow Discrimination of Rhesus NKG2C vs. NKG2A

Analysis of NKG2C vs.NKG2A expression on RM NK cells was carried out using RNA probe-based staining and flow cytometry (PrimeFlow # 88-18005-204, Affmetrix) as described (36). Briefly, KLRC1(NKG2A)-Alexa-647 and KLRC2(NKG2C)-Alexa-488 probe sets complementary to unique sequences in RM KLRC1 and KLRC2 mRNAs were purchased from Thermo Fisher (KLRC1 Assay ID VF1-20995-PF, KLRC2 Assay ID VF4-4221856-PF). Frozen PBMCs were thawed and rested in RPMI1640 with 10% FBS (Sigma, #F2442) at 37°C and 5% CO_2_ for ~12 h before staining. Surface marker antibody staining was performed followed by cell fixation and permeabilization for intracellular antibody and probe staining, using antibodies listed in Table S1. After staining and hybridization to probe sets, the cells were analyzed on the BD LSRFortessa II instrument.

### Computational and Statistical Analyses

R was used to realize the data and preform statistical analysis. Code can be found at https://github.com/dunbarlabNIH/CMV.

## Results

### Autologous Transplantation With Barcoded HSPC in RhCMV^neg^ Macaques

**Figure 1:**
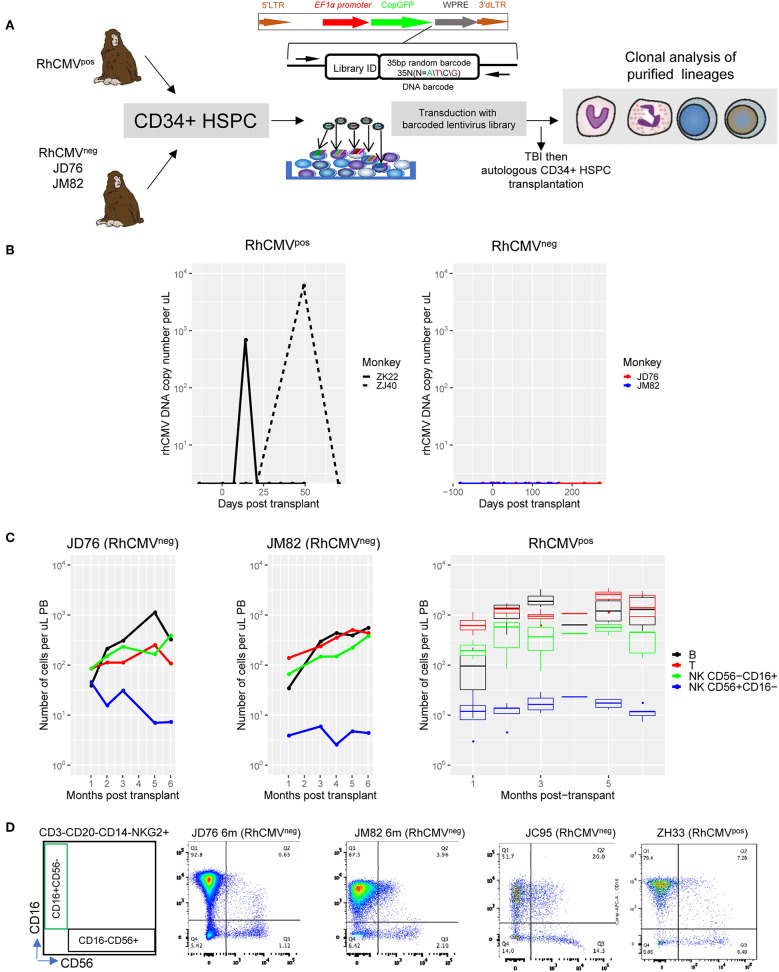
Experimental design and post-transplant reconstitution. **(A)** Pictorial representation of the experimental plan. The study includes five RhCMV^pos^ RM and two RhCMV^neg^ RM (JD76 and JM82) that were autologously transplanted with barcoded CD34^+^ HSPCs. One RhCMV^neg^ RM(JC95) was left untransplanted as normal blood donor. **(B)** RhCMV DNA in plasma of RhCMV^pos^ (ZK22 and ZJ40) and RhCMV^neg^ (JD76 and JM82) RMs before and post transplantations. The cell counts for B cells (black line), T cells (red), and CD56^−^CD16^+^ NK cells (green) and CD56^+^CD16^−^ NK cells (blue) over the first 6 months post-transplant for **(C)** RhCMV^neg^ and RhCMV^pos^ monkeys. The median and the 25–75% percentile range of cells count from 5 RhCMV^pos^(ZH33, ZG66, ZH19, ZJ31, and ZK22) for each lineage are shown. **(D)** FACS plots showing the CD16 and CD56 expression of CD3^−^CD20^−^CD14^−^NKG2^+^ NK cells for RhCMV^neg^ (JD76, JM82, and JC95) and a representative RhCMV^pos^ monkey (ZH33). The schematic of gating for sorts is shown on the left.

We utilized genetic barcoding of RM HSPC to study the impact of RhCMV infection on the clonal dynamics of NK and T cells following myeloablative autologous transplantation (Figure 1A). In this model, a high diversity library of barcodes is introduced into RM CD34^+^ HSPC, followed by total body irradiation (TBI) and autologous transplantation. Each individual barcode uniquely marks a HSPC and its progeny, and can be quantitatively retrieved to track the dynamics of thousands of barcoded clones (16, 18). RMs in our prior studies (*n* = 5) (16, 18) were all RhCMV seropositive, indicating prior infection and the presence of latent virus. Although serum and saliva were negative for detectable RhCMV DNA before myeloablative and highly immunosuppressive conditioning with high dose TBI, samples collected immediately following TBI and autologous transplantation showed evidence for RhCMV reactivation, before clearing 3–8 weeks later (Figure 1B). Three RhCMV^neg^ RM were obtained and two (JD76, JM82) underwent barcoded HSPC transplantation (Figure 1A, Figure S1A), with the third macaque (JC95) retained as a blood donor to support the other two animals following myeloablation until engraftment. As expected, RhCMV DNA was not detectable in either serum or saliva collected before or post-transplantation (Figure 1B, Figure S1B). RhCMV IgG remained negative post-transplantation. Both RhCMV^neg^ animals recovered neutrophil, red blood cell and platelet counts in the expected time frame (Figure S1C). Successful barcoded lentiviral vector transduction of engrafted HSPC was documented by detection of expression of the marker CopGFP gene at appreciable levels in engrafted circulating myeloid and lymphoid cells (Figures S1D,E).

To assess whether RhCMV status affects post-transplantation cellular immune reconstitution, we analyzed circulating numbers of T cells, B cells, CD56^+^CD16^−^ NK cells (analogous to human CD56^bright^ immature NK cells) and CD56^−^CD16^+^ NK cells (analogous to human CD56^dim^ mature NK cells) (13). RM NK cells were defined by expression of NKG2 using the anti-human NKG2A antibody which stains both NKG2A and NKG2C on RM NK cells (13, 38–40). There was no discernible difference in the pace or degree of recovery of these cell types post transplantation comparing RhCMV^neg^ to RhCMV^pos^ animals, including the mature CD56^−^CD16^+^ NK cells of most interest (Figure 1C). The distribution and staining pattern of CD56^+^CD16^−^ and CD56^−^CD16^+^ NK cells were also similar between RhCMV^neg^ and RhCMV^pos^ animals (Figure 1D).

### Clonal Dynamics in RhCMV^pos^ vs. RhCMV^neg^ Animals Post-transplantation

**Figure 2:**
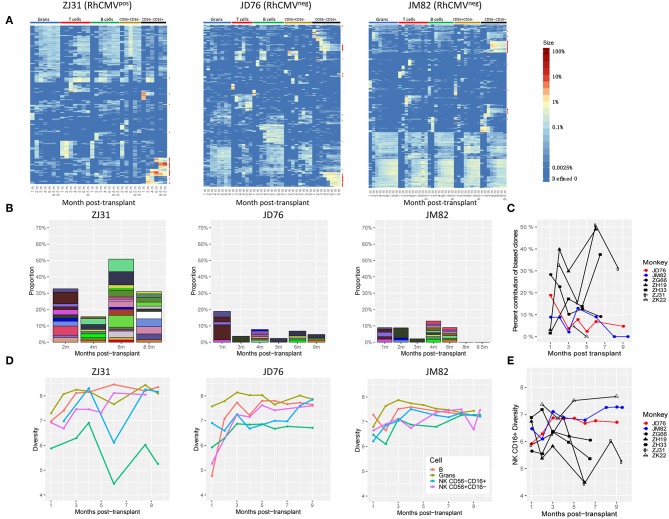
Clonal characterization of NK cells post-transplantation. **(A)** The upper panels show heatmaps of the top 10 barcoded clones chosen by rank order relative contributions to each sample for RhCMV^pos^ monkey ZJ31 and RhCMV^neg^ monkeys JD76 and JM82 over time post-transplantation. Each column shows a single sample and each row represents an individual barcode (clone). Contributions from the top 10 clones for each sample are plotted over all samples included in the analysis. Colors represent the relative percent contribution of the barcoded clone in that sample (column) as shown in the color bar on the right. Clones (rows) with at least 10-fold greater contribution to CD56^−^CD16^+^ NK cells (termed biased clones) than to any other lineage, including T cells, B cells, granulocytes and CD56^+^CD16^−^ NK cells are designated with red stars. **(B)** The stacked bar plots showing the fractional contribution of the biased CD56^−^CD16^+^ NK cell clones over time for RhCMV^pos^ monkey ZJ31 and RhCMV^neg^ monkeys JD76 and JM82 post transplantation. Each colored box represents a barcode clone. **(C)** The total percent contribution of CD56-CD16+ NK-biased clones for RhCMV^pos^ (black lines) and RhCMV^neg^ (red and blue lines) RM over time. **(D)** Shannon diversity plots of each lineage [T, B, Granulocytes(Grans), CD56^−^CD16^+^ NK and CD56^+^CD16^−^ NK] over time for RhCMV^pos^ ZJ31 and RhCMV^neg^ JD76 and JM82 post transplantation. **(E)** Shannon diversity of CD56^−^CD16^+^ NK cell lineage in various RM (black: RhCMV^pos^ monkeys, red and blue: RhCMV^neg^ monkeys).

As previously shown in our clonal tracking studies (16, 18), the CD56^−^CD16^+^ subset of NK cells is dominated by a limited number of very large barcoded clones highly biased in contributions toward only this NK subset, in comparison with polyclonal contributions from stable multilineage HSPC to all other circulating cell types appearing by 2–3 months post-transplant, as shown for RhCMV^pos^ ZJ31 in Figure 2 and for other RhCMV^pos^ animals in Figure S2. Individual clonal contributions can be visualized using heatmaps mapping the fractional contributions of individual barcodes, each corresponding to an individual clone derived from the same precursor (Figure 2A, Figure S2), and overall clonal diversity and richness for the entire population of clones can be represented via Shannon index plots (Figure 2A, Figure S2). In RhCMV^pos^ animals, mature circulating CD56^−^CD16^+^ NK cells are of much lower diversity than other lineages, and are primarily composed of expanded NK-biased clones that can wax and wane over time. Our previous work has demonstrated that these mature NK cell clones express specific KIR and likely self-renew and proliferate independent of ongoing production from HSPC (16, 18).

As shown in Figures 2A,B, CD56^−^CD16^+^ NK-biased clones arose in both RhCMV^neg^ macaques (JD76 and JM82) after engraftment. However, in comparison to RhCMV^pos^ animals, we observed that the CD56^−^CD16^+^ NK-biased clones were relatively smaller and in aggregate dropped to 10% or less of all clonal contributions in this cell type, in contrast to persistent contributions at levels of 10–50% from NK-biased clones in the RhCMV^pos^ animals (Figures 2B,C, Figure S2). In addition, the clonal diversity of CD56^−^CD16^+^ NK cells in the two RhCMV^neg^ recipients became similar to that of other lineages over time, in contrast to decreasing and generally markedly lower diversity in this cell population in RhCMV^pos^ animals (Figures 2D,E, Figures S2, S3). Taken together, the results indicate that biased peripherally-expanding NK clones are still generated post-transplantation in RhCMV^neg^ macaques, however they are less prominent than in RhCMV^pos^ animals, suggesting that RhCMV may be a major but not the only driver of mature NK clonal dynamics.

### Impact of RhCMV Infection on NK Cell Clonal Dynamics in RhCMV^neg^ Macaques

**Figure 3:**
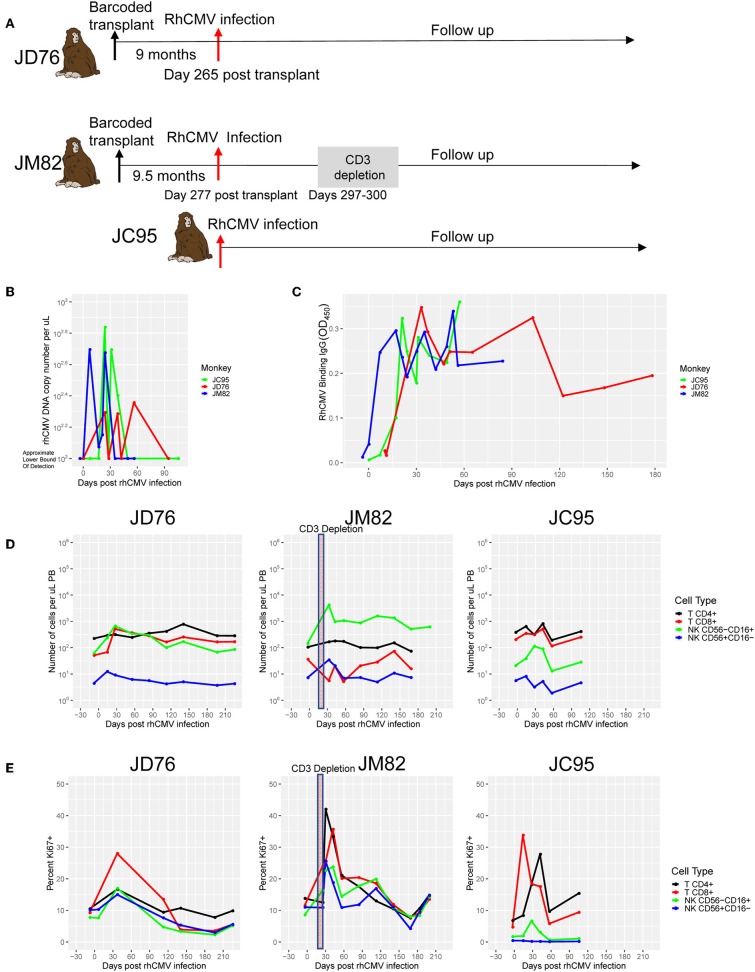
Experimental RhCMV infection. **(A)** Experimental design for RhCMV infection. RMs were infused with RhCMV 9 and 9.5-months post-transplantation. JM82 was T-depleted on days 20-23 post-infection (days 297–200 post-transplant). RhCMV plasma viral load and IgG serologies were followed over time and blood samples were collected for barcode analysis. **(B)** RhCMV DNA copy number detection in plasma post-infection. The lower limit of detection is estimated by the number of copies obtained that received a non-empty read from the qPCR machine on two of three replicates. **(C)** Anti-RhCMV IgG in plasma post-infection for the three investigated RMs. **(D)** Cell counts per ul post-infection for CD4+ and CD8+ T cells and CD56^−^CD16^+^ and CD56^+^CD16^−^ NK cells. **(E)** The Ki67^+^ percentages in each cell population over time before and after RhCMV infection. The shaded boxes on JM82 plots in panel D and E indicate CD3^+^ T depletion.

To directly analyze the NK response to RhCMV, we infected RhCMV^neg^ barcoded macaques JD76 and JM82 with RhCMV strain 180.92 (28) 9–9.5 m post-transplantation. In addition, we also infected the non-transplanted RhCMV^neg^ macaque JC95 (Figure 3A). In JM82, CD3^+^ T cells were depleted following RhCMV administration in an attempt to reduce competition for proliferative cytokines by T cells. In all three macaques, productive infection was detected by 7–10 days after administration (Figure 3B, Figure S4A). After RhCMV DNA disappeared from the plasma around 60 days post infection, it remained undetectable, however small amounts of RhCMV DNA could found intermittently over time in saliva and urine, consistent with the normal pattern of virus shedding (Figure S4A). All three animals seroconverted between days 7 and 21(Figure 3C).

Following RhCMV administration, we enumerated circulating CD4^+^ and CD8^+^ T cells and CD56^+^CD16^−^ and CD56^−^CD16^+^ NK cells (Figure 3D), B cells (Figure S4B), and monocytes (Figure S4B), and the fraction of proliferation in each cell type by Ki67 staining (Figure 3E). In JD76, the numbers of CD8^+^ T cells and both NK cell subsets increased markedly after infection, coincident with increased proliferation. In JM82, administration of the anti-CD3 immunotoxin delayed the initial increase in CD8^+^ T cells, accompanied, as expected, by more marked initial proliferation of NK cells. In untransplanted JC95, T cells and CD56^−^CD16^+^ NK cells increased following infection. Overall, there was a rapid and marked immune response to RhCMV infection in all compartments. Of note, we also observed a second increase in cell numbers and proliferation of T and NK subsets at 4-5m post RhCMV infection in all three animals (Figures 3D,E).

**Figure 4:**
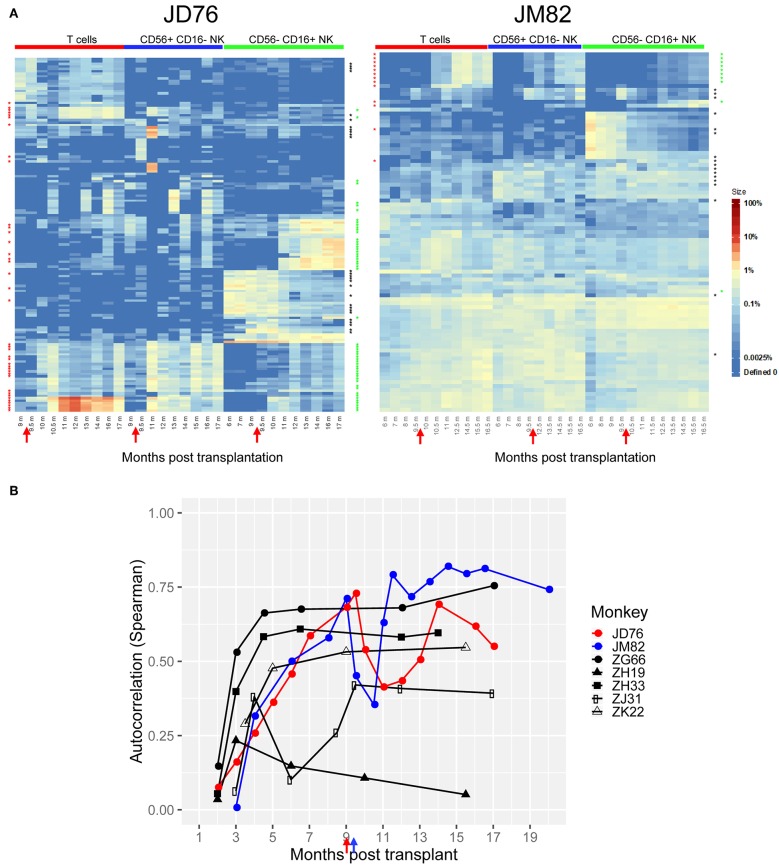
Impact of RhCMV infection on immune cell clonality. **(A)** Heatmaps displaying the contributions from the top 10 clones from each sample plotted over time in animals JD76 and JM82 before and after RhCMV administration, constructed as explained in the legend to Figure 2. The red arrows on the x axis show when RhCMV was administered in relation to months post-transplantation. The color scale for fractional clonal contributions is shown on the right. Red stars to the left of each heat map designate barcodes (clones) that increased fractional contributions >10 fold to T cells between the pre-RhCMV time point and post-RhCMV time points. Green stars to the right of each heat map designate barcodes that increased fractional contributions >10 fold to CD56^−^CD16^+^ NK cells between the pre-RhCMV time point and post-RhCMV time points. Black stars to the right of each heat map designate barcodes that decreased fractional contributions >10 fold to CD56^−^CD16^+^ NK cells between the pre-RhCMV time point and post-RhCMV time points. **(B)** Autocorrelation plots display the Spearman correlation between clonal contributions to adjacent time points in CD56^−^CD16^+^ NK cells. Samples with close to identical clonal contributions will have an autocorrelation close to 1 and very dissimilar clonal contributions will have an autocorrelation near 0. RhCMV^pos^ RM are shown in black and the two barcoded RhCMV^neg^ RM are shown in red(JD76) and blue(JM82). The autocorrelation between two time points is plotted at the later of the two time points being compared. The arrows in red(JD76) and blue(JM82) on the bottom of X axis indicate the time of RhCMV infection.

We followed the clonal patterns over time in each lineage before and after RhCMV infection. As shown in Figure 4A, JD76 developed a very prominent T cell clonal response, with a number of T cell clones becoming dominant and strongly biased toward the T cell lineage, implying peripheral expansion, beginning at about 1-month post-infection (red stars in Figure 4A), coinciding with the proliferation and increase in total T cells observed in this compartment (Figures 3D,E). We confirmed that these expanding T cell clones were primarily CD8^+^ T cells (Figure S4C). In contrast, following peri-infection T depletion in JM82, no marked changes in T cell clonality were observed at 1 month post-infection; however, groups of enlarging T cell clones did appear 3 months post RhCMV infection in JM82, upon later regeneration of the T cell compartment (Figure 4A, red stars on the left y axis).

In CD56^−^CD16^+^ NK cells following RhCMV infection, we did not observe any immediate (<1 month post infection) marked changes in the clonal contributions in JD76 examining the largest contributing clones on heatmap analyses (Figure 4A) despite an increase in NK CD56^−^CD16^+^ cell numbers in the PB (Figure 3D). No trackable clones increased in relative contribution more than 10-fold during the first month post-infection. Thus, the rapid proliferation and increase in numbers of circulating mature NK cells immediately following infection in this animal appeared to result from a polyclonal, clonally non-specific response. However, at 2 m post-infection (11 m post-transplantation), when mature NK numbers had stabilized, the clonal profile markedly changed, with multiple new NK-biased clones expanding in relative contribution and persisting for up to 8 m post-infection (green stars on the right of heatmaps in Figure 4A) and a relative decrease in contributions from some large clones present before infection (black stars in Figure 4A). These clonal shifts could have resulted from preferential proliferation or enhanced survival of specific expanding clones and/or exhaustion or differential contraction of the disappearing NK clones. The mature NK clonal pattern in JM82 (T cell depletion) also showed disappearance of a set of biased NK clones beginning 0.5 m following infection (black stars) and expansion of a new set of small clones beginning 2 m post-infection and persisting (green stars) (Figure 4A), but most of the marked proliferation and increase in NK numbers early after infection in this animal appears to have resulted from a non-specific, pan-clonal stimulation.

We analyzed autocorrelations (Spearman) of all clonal contributions to CD56^−^CD16^+^ NK over time (Figure 4B), comparing relatedness of clonal patterns to the immediately preceding sample. The RhCMV^pos^ animals overall showed relative stability by 3–6 months post-transplant, indicating slow and steady clonal modulations, other than one marked change at 6 months in ZJ31. However, in both RhCMV^neg^ animals, autocorrelations sharply dipped following RhCMV infection (marked by arrows Figure 4B), indicating shifts in clonal composition between adjacent time points. While these shifts could have occurred for other reasons, the timing is very suggestive for a link between the clonal shifts and RhCMV infection. These analyses suggest that RhCMV infection did significantly impact on mature NK clonal dynamics, resulting in exhaustion of some clones and relative expansion and persistence of new clones.

### Clonal Pattern Following Re-infection With RhCMV

**Figure 5:**
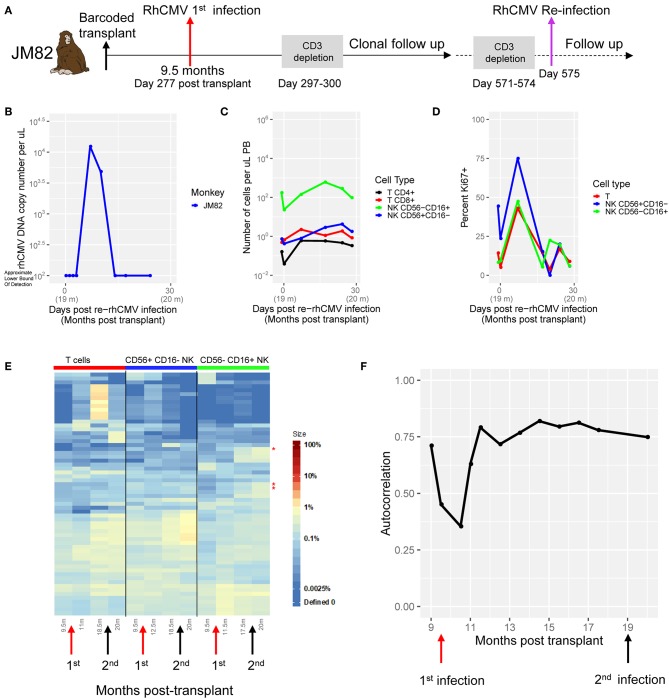
RhCMV Reinfection. **(A)** Experimental design. Reinfection of JM82 at day 298 post-initial infection (day 575 post-transplant). T cells were depleted day 294–297 post-initial infection (571–574 post-transplant). **(B)** RhCMV DNA copies in plasma post-reinfection. **(C)** Cell counts of CD4^+^ and CD8^+^ T cells and CD56^−^CD16^+^ and CD56^+^CD16^−^ NK cells post-reinfection. **(D)** Ki67^+^ percent in each lineage post-reinfection. **(E)** Heatmap for each lineage pre and post-infection. Colors represent percent contribution to a sample. Stars indicate NK cells that are 2 times expanded from baseline. **(F)** Autocorrelation plots display the correlation (spearman) between adjacent time points of the CD56^−^CD16^+^ NK lineage.

We studied potential adaptive/memory responses to RhCMV by re-inoculating JM82 with RhCMV at 10 months post the first RhCMV infection (at day 575, or 19.5 m post transplantation), immediately following T cell depletion (Figure 5A), resulting in clear but short-lived viremia (Figure 5B). Not surprisingly, RhCMV was cleared much more quickly than following the initial infection(9 days vs. 57 days post infection) (Figures 3B, 5B). Both CD56^−^CD16^+^ and CD56^+^CD16^−^ NK cells again expanded and proliferated following re-infection (Figures 5C,D), with some increase in T cell numbers and marked residual T cell proliferation (Ki67^+^) despite anti-CD3 immunotoxin administration. Notably, in the post-reinfection samples, the peak Ki67 percentage in CD56^−^CD16^+^ NK cells was much higher than post-initial infection (~50 vs. 25%). We analyzed the clonal pattern following re-infection, and did not observe any major new clonal shifts, as analyzed by both clustering heat map analysis (Figure 5E) and autocorrelation analysis (Figure 5F), other than 3 defined expanded CD56^−^CD16^+^ NK clones appeared at 1 m post-reinfection from the existing clones (red stars on the right of heatmap, Figure 5E).

### Increase in NKG2C^+^ CD16^+^ NK Cells Following RhCMV Infection

**Figure 6:**
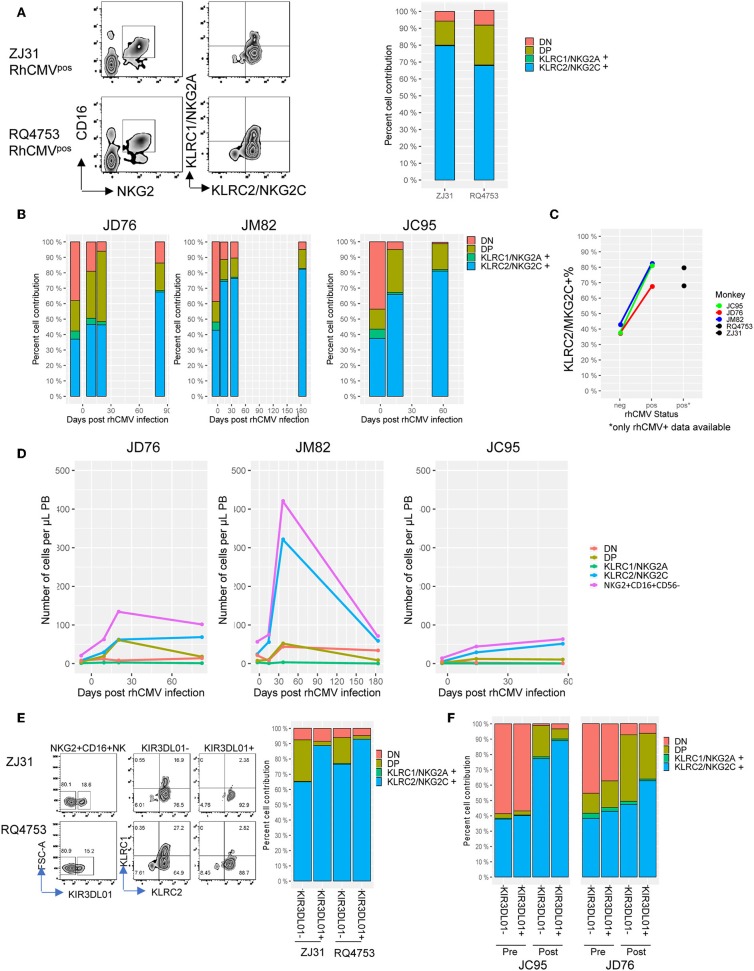
Analysis of NKG2A vs. NKG2C transcript expression in CD56-CD16+ NK cells. **(A)** FACS plots for KLRC1 (NKG2A) and KLRC2 (NKG2C) RNA analysis for RhCMV^pos^ animals (barcode monkey ZJ31 and untransplanted RQ4753). The right panel shows a barplot for the two animals where the percent KLRC1/NKG2A^+^, KLRC2/NKG2C^+^ double positive (DP) and double negative (DN) CD56^−^CD16^+^ NK cells is plotted. **(B)**The NKG2A/C profile in CD56^−^CD16^+^ NK cells over time before and post RhCMV infection. **(C)** Comparison of the percent NKG2C positive CD56^−^CD16^+^ NK cells in RhCMV^neg^ and RhCMV^pos^ animals. Lines connect the pre-infection and latest post-infection sample for the initially RhCMV^neg^ animals, and RhCMV^pos^ animals are shown as black points. **(D)** Absolute cell numbers for each cell population in the PB post initial infection over time from JD76, JM82, and JC95. Total CD56^−^CD16^+^ NK cell numbers are shown in purple. **(E)** left panels show the FACS plots for the expression of KIR3DL01 and KLRC1/KLRC2 on NKG2^+^ CD56^−^CD16^+^ NK cells from two RhCMV^pos^ monkeys. Right panel shows the a barplot for the two animals where the percent contribution to CD56^−^CD16^+^KIR3DL01^+^ and CD56^−^CD16^+^KIR3DL01^−^ NK subpopulations is plotted for KLRC1/NKG2A^+^, KLRC2/NKG2C^+^, double positive (DP) and double negative (DN) cells. **(F)** Barplots for the two RhCMV^neg^ animals (JC95 and JD76) where the percent contribution to CD56^−^CD16^+^KIR3DL01^+^ and CD56^−^CD16^+^KIR3DL01^−^ NK subpopulations is plotted for KLRC1/NKG2A^+^, KLRC2/NKG2C^+^, double positive (DP) and double negative (DN) cells, samples from pre- and post RhCMV infection are shown.

It has been hypothesized in the literature that NKG2C, an activating receptor, is a marker of NK cell adaptive responses in humans (22, 41), in contrast to NKG2A, the inhibitory isoform (42). Given the lack of antibodies that discriminate between the two isoforms in macaques, RNA probe single cell staining and analysis by FACS was recently shown to be a feasible alternative methodology for analysis of RM NK cells, and RhCMV^pos^ macaques were shown to have a higher fraction of NKG2C^+^ NK cells than RhCMV^neg^ macaques (36). Using this approach, we analyzed NKG2C and NKG2A expression in CD56^−^CD16^+^ NK cells in two RhCMV^pos^ macaques (Figure 6A), as well as in samples before and after RhCMV infection in the three RhCMV^neg^ macaques (Figure 6B). The three RhCMV^neg^ macaques greatly increased the fraction and absolute number of NKG2C^+^ NK cells (Figures 6B–D), accounting for almost all of the increase in CD56^−^CD16^+^ NK cells in these animals following infection. This proportion stabilized or continued to increase over time post-infection up to 6 m post RhCMV infection. Of note, the fraction and absolute number of NKG2A/NKG2C double positive cells increased before the maximal level of NKG2C^+^ cells in all three macaques, consistent with a model of transit through a double positive state before final maturation to NKG2C^+^ adaptive NK cells (22, 36).

Human adaptive NK cells often express both inhibitory and activating killer immunoglobulin-like receptors (KIR) and NKG2C (10, 11). We stained the NK cells with both an anti-human KIR2D (clone NKVFS1) antibody which recognizes the rhesus KIR3DL01 (43, 44) and the NKG2C probes. As shown in Figure 6E, in the RhCMV^pos^ monkeys ZJ31 and RQ4753, more than 88% of the KIR3DL01^+^ NK cells express NKG2C as detected by KLRC2 probe. In both monkeys, within the KIR3DL01^−^ NK population, the fraction of cells expressing NKG2C is lower than in the KIR3DL01^+^ NK population. In the 3 RhCMV^neg^ monkeys, two (JD76 and JC95) express KIR3DL01^+^ on a fraction of their CD56^−^CD16^+^ NK cells. Both KIR3DL01 positive and negtive NK cells increased NKG2C expression to a similar degree following CMV infection in JC95. In contrast in JD76, there appeared to be differential expansion of NKG2C^+^ cells expressing this particular KIR following RhCMV infection, suggesting this KIR potentially could interact specifically with RhCMV (Figure 6F).

## Discussion

We previously reported oligoclonal expansions of mature macaque NK cells appearing rapidly following transplantation, maintained independently of ongoing maturation from HSPC, and clonally-segregating with expression of specific KIR (18). In the current study, we hypothesized that RhCMV, reactivated following transplantation, might be driving the expansion and persistence of these expanded, long-lived NK clones. The overall pattern of NK cell recovery and phenotype following transplantation was similar in RhCMV^pos^ and RhCMV^neg^ animals. Expanded, NK-biased CD16+ mature NK clones still appeared in the two transplanted RhCMV^neg^ macaques following engraftment, however the size of individual clones and overall contributions appeared to be smaller than observed in the majority of RhCMV^pos^ macaques.

These observations provide some support for the hypothesis that RhCMV does play a role in the stimulation and maintenance of these expanded and persistent mature NK cell clones, but the appearance of CD56^−^CD16^+^ expanded, NK-biased clones even in the CMV^neg^ animals suggest that other stimuli must contribute to their manifestation. We speculate that additional latent herpes viruses, such as lymphocryptoviral (LCV), the RM equivalent of Epstein Barr Virus (EBV), may be stimulating NK cell clonal expansions due to reactivation post transplantation. The two RhCMV^neg^ animals we transplanted in this study were from “specific pathogen free” colonies, defined as negative for tuberculosis, herpes B virus, type D simian retrovirus, STLV1 (simian equivalent of HTLV1), and SIV (the simian equivalent of HIV), but were both serologically LCV^pos^ at the time of transplantation. EBV reactivation occurs frequently following human HSPC autologous transplantation (45, 46). While recent work focuses primarily on the link between CMV and NK cells with adaptive properties, other studies have linked EBV to mature NK cell responses. For example, it was observed in the study that human CMV^pos^ students acquiring acute EBV infection expanded CD56^dim^ NK cells, albeit expressing the inhibitory receptor NKG2A, not the activating receptor NKG2C associated with CMV infection (47). A recent publication analyzed hematopoietic clonal diversity via insertion site retrieval in children with adenosine deaminase-deficient severe combined immunodeficiency treated with retroviral gene therapy, revealing massive expansion of a CD56^dim^ NK cell clone coincident with EBV reactivation post- transplantation (48).Other reports also suggest important roles for NK cells in response to EBV in human (49, 50). Whether EBV plays role in inducing mature clonal NK expansion would benefit from further investigation in our barcode model (16, 51).

When we experimentally induced primary RhCMV infection in the two barcoded RhCMV^neg^ macaques 9–10 months transplantation, we observed proliferation of T and NK cells in the PB at 0.5–1 months post-infection and an expansion in circulating cell numbers. During this acute phase, coinciding with viremia, clonal patterns in both T and NK cells in terms of relative contributions from individual clones did not markedly change, suggesting a non-specific homogeneous expansion/proliferation of these compartments in response to inflammatory stimuli such as cytokines. 1–2 months following infection, new expansions of both T cell clones and mature CD56^−^CD16^+^ NK cell clones appeared. Although we observe clear changes in the clonal profile following infection and reinfection, it is difficult to discern if the changes observed are due to specific NK responses to RhCMV infection, given the observation of waxing and waning NK expanded clones in both RhCMV^pos^ and RhCMV^neg^ animals. However, the autocorrelation analyses presented in Figure 4 suggest a marked specific change beyond underlying clonal fluctuations occurring following RhCMV primary infection in the RhCMV^neg^ animals. In addition, very rapid clearance of a second RhCMV challenge 9 months after the initial infection occurred coincident with CD56^−^CD16^+^ NK cell proliferation, without major clonal NK shifts, suggesting long-term persistence of the NK RhCMV reactive clonal repertoire. It could be possible that the NK cells expansion is stimulated by other proliferating cell populations such as T cells post RhCMV infection, however, with T cells depletion in JM82 at the time of first RhCMV infection and prior to the second RhCMV infection, we still observed obvious CD56^−^CD16^+^ NK expansions post each RhCMV infection.

The lack of antibodies able to distinguish NKG2A from NKG2C expression on RM NK cells (40) has hindered direct comparisons between putative human adaptive NK cell responses to RhCMV infection or reactivation, characterized by expansion of NKG2C^+^ mature NK cells in multiple studies (21, 22, 47). Using RNA probes able to distinguish expression of the two genes by flow cytometry (36), as previously used to uncover higher fractions of NKG2C-expressing cells in RhCMV^pos^ vs. RhCMV^neg^ animals, we documented increase expression in CD16^+^ NK cells in the three animals we monitored before and after infection, further supporting the relevance of our model to NK dynamics in humans following RhCMV infection. We also observed that about 90% of the KIR3DL01^+^CD16^+^ NK cells were NKG2C^+^ in both RhCMV^pos^ and RhCMV^neg^ monkey post infection, this results strongly links these two adaptive memory markers together to provide further evidences for NK adaptive immune features.

In conclusion, by studying RhCMV^neg^ animals and subsequently infecting them in a rhesus macaque model allowing tracking of individual NK cell clones, we have shown long-lasting clonal expansions arising in response to RhCMV, suggesting a clonal adaptive response with the potential to retain immunological memory. These analyses raise additional questions regarding NK dynamics in response to environmental cues with relevance to clinical adoptive NK cell transfer which we will examine in future barcoding experiments in the rhesus macaque model.

## Data Availability Statement

The datasets analyzed for this study can be found in github.com (https://github.com/dunbarlabNIH/CMV).

## Ethics Statement

The animal study was reviewed and approved by the NIH Animal Care and Use Committee (ACUC).

## Author Contributions

Conceptualization: CW, CD, AK, LT, and DY. Analytics and statistical analyses: LT and DE. Investigation: LT, DY, DE, XF, DR, MM, DT, LS, and CW. Resources: AK, RR, and RD. Writing: LT, CD, and CW. Supervision: AK, CD, and CW.

### Conflict of Interest

The authors declare that the research was conducted in the absence of any commercial or financial relationships that could be construed as a potential conflict of interest.
