# Supplementary material for: Impact of CMV Infection on Natural Killer Cell Clonal Repertoire in CMV-Naïve Rhesus Macaques
Source: Front Immunol. 2019 Oct 9;10:2381. doi: 10.3389/fimmu.2019.02381 (PMC6794559; doi:10.3389/fimmu.2019.02381)
Supplement: Supplementary file 1 [file Data_Sheet_1.PDF]

Table S1: The information of antibodies

Antigen	Conjugation	Vendor	Catalog number	Clone
CD3	APC-Cy7	BD Pharmingen	557757	SP34-2
CD3	V500	BD Pharmingen	560770	SP34-2
CD20	PE-Cy5	BD Pharmingen	555624	2H7
CD20	APC-Cy7	BD Pharmingen	335794	L27
CD14	Pacific Blue	Invitrogen	MHCD1428	TuK4
CD14	APC-Cy7	Biolegend	301820	M5E2
CD16	APC	Biolegend	302012	3G8
CD16	PE-Cy7	BD Pharmingen	557744	3G8
CD56	PE	BD Pharmingen	555516	B159
CD56	PE-Cy5	BD Pharmingen	555517	B159
NKG2A	APC	Beckman Coulter	A60797	Z199
Ki67	BV421	BD Pharmingen	562899	B56
NKP80	APC	Miltenyi Biotec	130-094-845	N/A
CD8	BV711	Biolegend	301044	RPA-T8
KIR2D	PE	Miltenyi Biotec	130-092-688	NKVFS1
CD11c	PE Cy7	BioLegend	301608	3.9
HLA-DR	PE-Texas Red	Invitrogen	MHLDR17	TU36
IFNg	APC	BD Pharmingen	554702	B27
TNF	PE	Biolegend	502909	MAb11
CD107a	Pacific Blue	Biolegend	328624	H4A3
CD16	purified	Biolegend	302002	3G8
Live/dead	Aqua dye	Life technology	L34957	N/A

Table S2: Sequences of primers and probes

Primer Names	5’-----------3’ sequences	Index
Universal reverse primer	CAAGCAGAAGACGGCATACGAGATCGTGATACGGCATACGAGCTCTTCCGATCT	
Barcode New Forward i501	AATGATACGGCGACCACCGAGATCTACACTCGCCTTAACACTCTTTCCCTACACGACGCTCTTCCGATCT	TCGCCTTA
Barcode New Forward i502	AATGATACGGCGACCACCGAGATCTACACCTAGTACGACACTCTTTCCCTACACGACGCTCTTCCGATCT	CTAGTACG
Barcode New Forward i503	AATGATACGGCGACCACCGAGATCTACACTTCTGCCTACACTCTTTCCCTACACGACGCTCTTCCGATCT	TTCTGCCT
Barcode New Forward i504	AATGATACGGCGACCACCGAGATCTACACGCTCAGGAACACTCTTTCCCTACACGACGCTCTTCCGATCT	GCTCAGGA
Barcode New Forward i505	AATGATACGGCGACCACCGAGATCTACACAGGAGTCCACACTCTTTCCCTACACGACGCTCTTCCGATCT	AGGAGTCC
Barcode New Forward i506	AATGATACGGCGACCACCGAGATCTACACCATGCCTAACACTCTTTCCCTACACGACGCTCTTCCGATCT	CATGCCTA
Barcode New Forward i507	AATGATACGGCGACCACCGAGATCTACACGTAGAGAGACACTCTTTCCCTACACGACGCTCTTCCGATCT	GTAGAGAG
Barcode New Forward i510	AATGATACGGCGACCACCGAGATCTACACCAGCCTCGACACTCTTTCCCTACACGACGCTCTTCCGATCT	CAGCCTCG
Barcode New Forward i511	AATGATACGGCGACCACCGAGATCTACACTGCCTCTTACACTCTTTCCCTACACGACGCTCTTCCGATCT	TGCCTCTT
Barcode New Forward i512	AATGATACGGCGACCACCGAGATCTACACTCCTCTACACACTCTTTCCCTACACGACGCTCTTCCGATCT	TCCTCTAC
Barcode New Forward i514	AATGATACGGCGACCACCGAGATCTACACTCATGAGCACACTCTTTCCCTACACGACGCTCTTCCGATCT	TCATGAGC
Barcode New Forward i515	AATGATACGGCGACCACCGAGATCTACACCCTGAGATACACTCTTTCCCTACACGACGCTCTTCCGATCT	CCTGAGAT
Barcode New Forward i516	AATGATACGGCGACCACCGAGATCTACACTAGCGAGTACACTCTTTCCCTACACGACGCTCTTCCGATCT	TAGCGAGT
Barcode New Forward i518	AATGATACGGCGACCACCGAGATCTACACGTAGCTCCACACTCTTTCCCTACACGACGCTCTTCCGATCT	GTAGCTCC
Barcode New Forward i519	AATGATACGGCGACCACCGAGATCTACACTACTACGCACACTCTTTCCCTACACGACGCTCTTCCGATCT	TACTACGC
Barcode New Forward i520	AATGATACGGCGACCACCGAGATCTACACAGGCTCCGACACTCTTTCCCTACACGACGCTCTTCCGATCT	AGGCTCCG
Barcode New Forward i521	AATGATACGGCGACCACCGAGATCTACACGCAGCGTAACACTCTTTCCCTACACGACGCTCTTCCGATCT	GCAGCGTA
Barcode New Forward i522	AATGATACGGCGACCACCGAGATCTACACCTGCGCATACACTCTTTCCCTACACGACGCTCTTCCGATCT	CTGCGCAT
Barcode New Forward i523	AATGATACGGCGACCACCGAGATCTACACGAGCGCTAACACTCTTTCCCTACACGACGCTCTTCCGATCT	GAGCGCTA
Barcode New Forward i524	AATGATACGGCGACCACCGAGATCTACACCGCTCAGTACACTCTTTCCCTACACGACGCTCTTCCGATCT	CGCTCAGT
Barcode New Forward i526	AATGATACGGCGACCACCGAGATCTACACGTCTTAGGACACTCTTTCCCTACACGACGCTCTTCCGATCT	GTCTTAGG
Barcode New Forward i527	AATGATACGGCGACCACCGAGATCTACACACTGATCGACACTCTTTCCCTACACGACGCTCTTCCGATCT	ACTGATCG
Barcode New Forward i528	AATGATACGGCGACCACCGAGATCTACACTAGCTGCAACACTCTTTCCCTACACGACGCTCTTCCGATCT	TAGCTGCA
Barcode New Forward i529	AATGATACGGCGACCACCGAGATCTACACGACGTCGAACACTCTTTCCCTACACGACGCTCTTCCGATCT	GACGTCGA
rhCMV Forward	TGCGTACTATGGAAGAGACAATGC	
rhCMV Reverse	ACATCTGGCCGTTCAAAAAAAC	
rhCMV Probe	56-FAM/CCGAAGTT/ZEN/GCGCATCCGCTTGT/3IABkGQ	

A

	ZH33	ZG66	ZJ31	ZK22	ZH19	JD76	JM82	JC95
RhCMV status at transplantation	positive	positive	positive	positive	positive	negative	negative	negative
% GFP^{+} infused cells	35	35	35	31	23	28	34	N/A
$CD34^{+}$ HSPC dose (millions)	32	48	23	82	48	44	91	N/A
CD34+ transplant dose/kg (millions)	6.9	8.5	4.1	7.2	7.1	4.1	7.2	N/A
Infused GFP^{+} cells (millions)	11.1	16.7	8	25.2	11	12.3	30.6	N/A
KIR3DL05 (detected by Tetramer GY9/Mamu-A1*002)	-	-	-	+	+	+	+	+
KIR3DL01 (detected by Anti-KIR2D (NKVFS1,human))	+	+	+	-	+	+	-	+

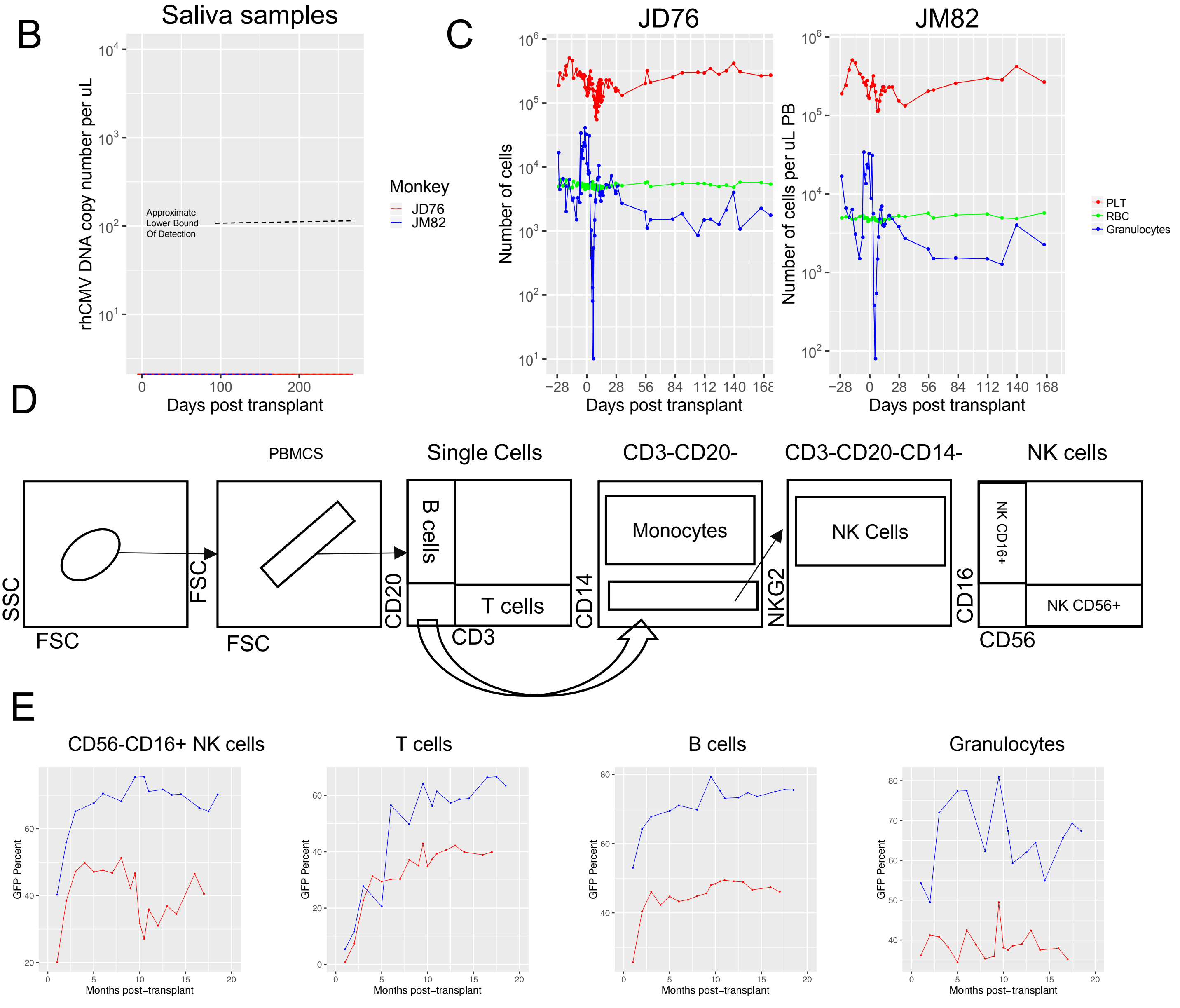

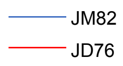

Figure S1

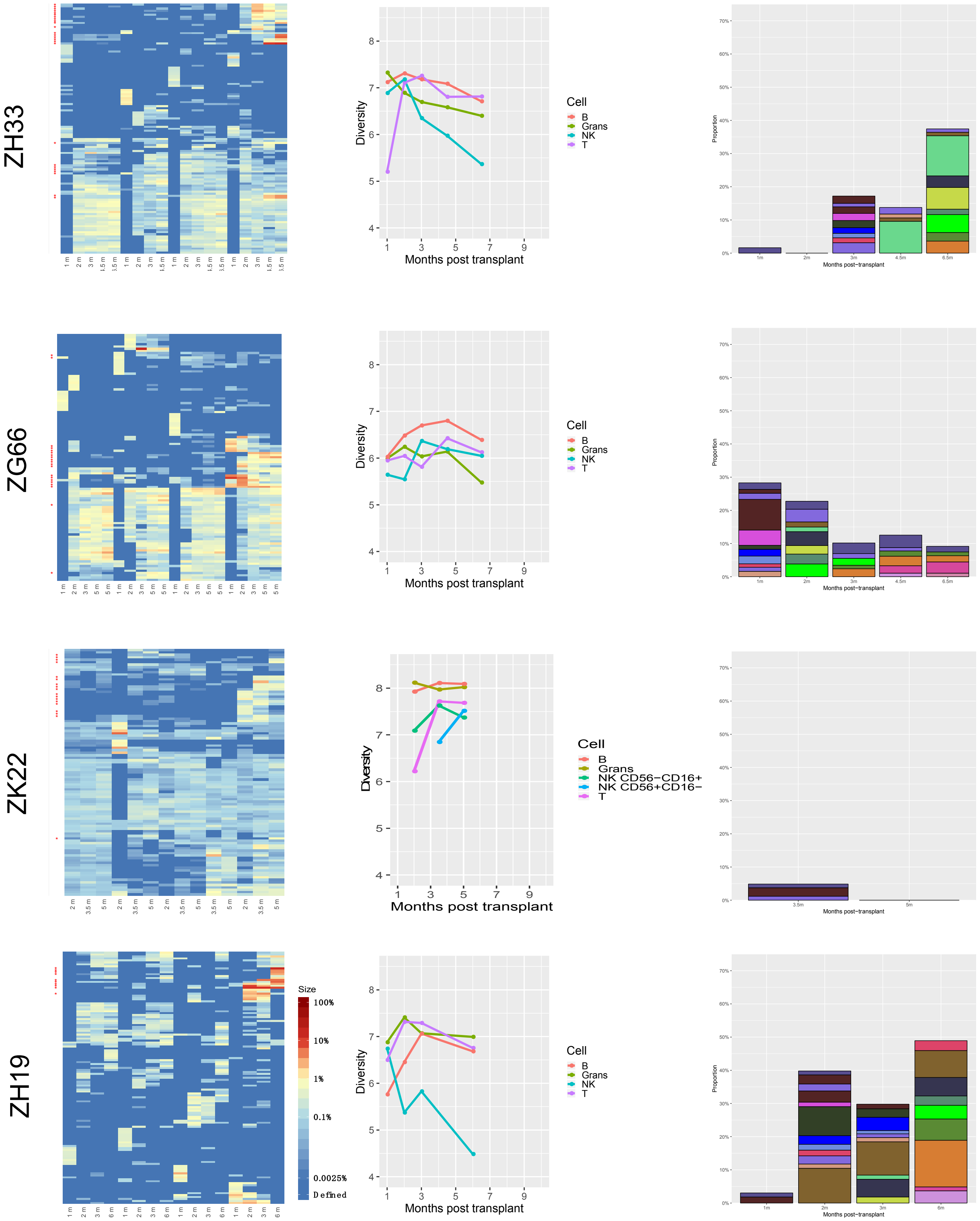

ZH33
ZG66
ZK22
ZH19
Diversity
Months post transplant
Cell
B
Grans
NK
T
NK CD56−CD16+
NK CD56+CD16−
Size
100%
10%
1%
0.1%
0.0025%
Defined
Proportion
Months post−transplant

Figure S2

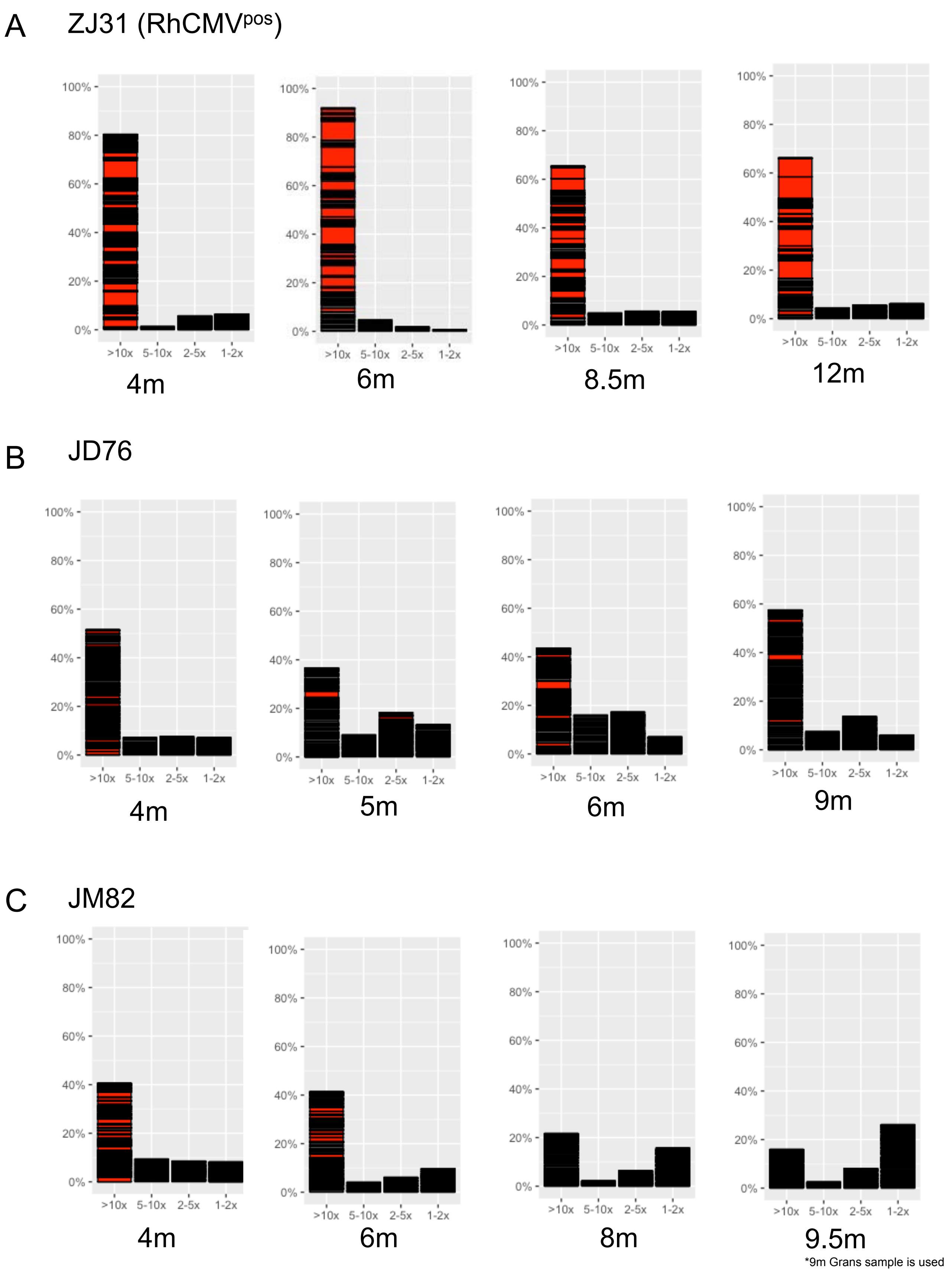

A ZJ31 (RhCMV^pos)
B JD76
C JM82
100%
80%
60%
40%
20%
0%
>10x
5-10x
2-5x
1-2x
4m
5m
6m
8m
8.5m
9m
9.5m
12m
*9m Grans sample is used

Figure S3

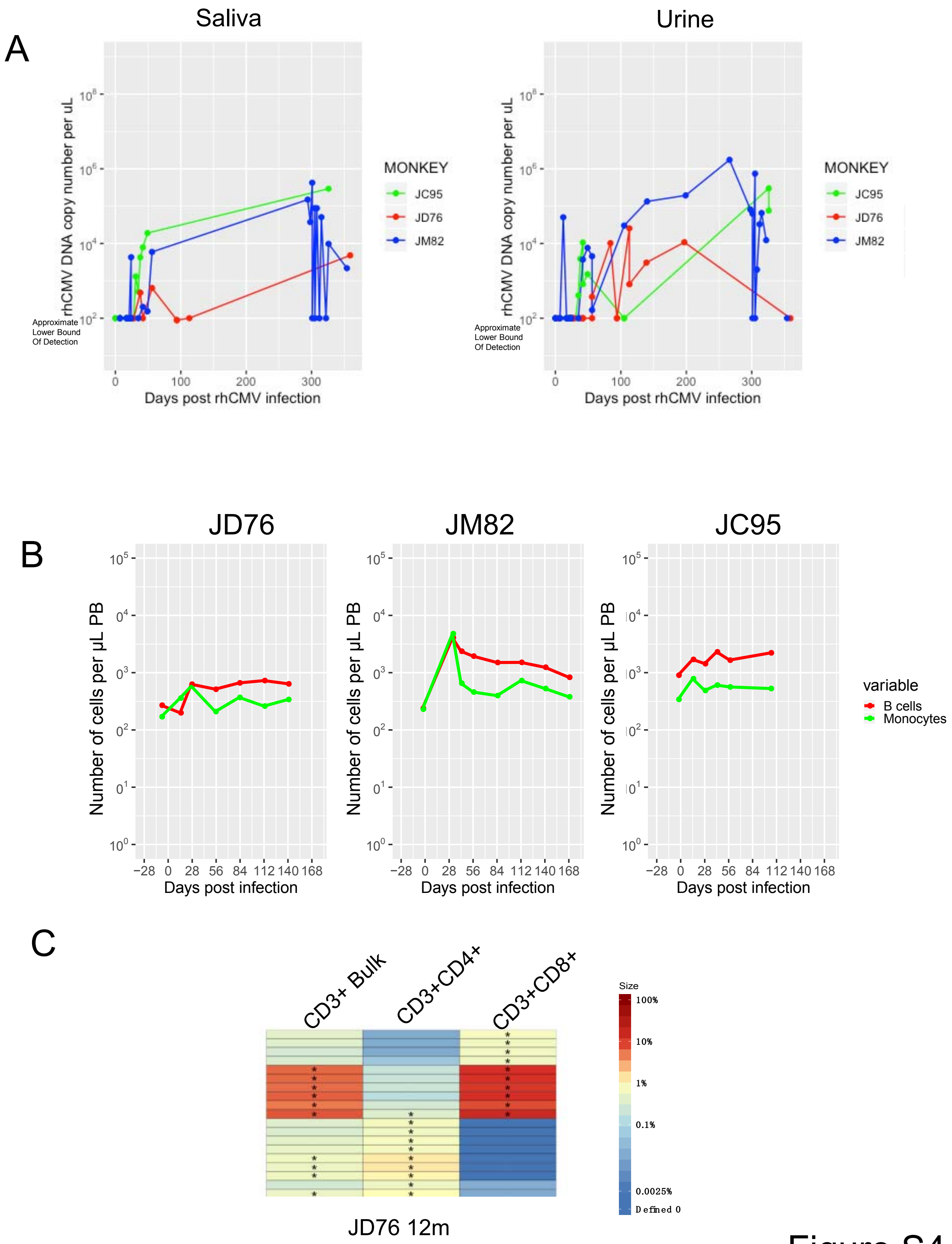
A
Saliva
Urine
rhCMV DNA copy number per uL
Approximate Lower Bound Of Detection
Days post rhCMV infection
MONKEY
JC95
JD76
JM82
B
JD76
JM82
JC95
Number of cells per µL PB
Days post infection
variable
B cells
Monocytes
C
CD3+ Bulk
CD3+CD4+
CD3+CD8+
Size
100%
10%
1%
0.1%
0.0025%
Defined 0
JD76 12m

Figure S4

Supplemental Figure 1

(A) Table of transplantation information and KIR haplotypes of the RM used in this study. (B) RhCMV DNA in saliva of RhCMV^{neg} (JD76 and JM82) RMs before and post transplantations. (C) Platelets, red blood cells, and neutrophil counts for JD76 and JM82 post-transplant. (D) Classic gating scheme for sorted cell types. Major populations are shown as blue rectangles/ovals.(E) GFP marker gene expression for various cell types of interest post-infection for the two transplanted RhCMV^{neg} animals.

Supplemental Figure 2

Post-transplant figures for additional RhCMV^{pos} animals. On the left are heatmaps. Color represents percent contribution in a particular sample. Samples are columns and barcodes are rows. When NK cells were sorted by CD56 and CD16, the heatmaps order from grans, T cells, B cells, NK CD56+CD16- cells, to NK CD56-CD16+. When not available, cells are ordered from grans, T cells, B cells, to NK cells. Red stars on the left of plots label clones that are 10x expanded in NK cell lineages compared to other lineages, regardless of percent contribution. In the middle are Shannon Diversity indexes for various cell types over time. When possible NK CD56-CD16+ and NK CD56+CD16- cells are differentiated, but this is not always possible. On the right are barplots of NK CD56-CD16+ or bulk NK biased clones. Bias is defined by total percent contribution >1% and 10x greater contribution than in any other lineage. In order to keep consistency, all plots limit results to the first 9 months post-transplant.

Supplemental Figure 3

Bias plots for animals post-transplant. (A) RhCMV^{pos} RM ZJ31, (B) JD76, (C) JM82. We took all barcodes that were biased in any amount towards NK cells i.e. their percent contribution was highest in NK cells compared to other lineages. On the x-axis is the binned amount of bias. For example, in the 2-5x bin are all barcodes that are between 2 and 5 times higher in

NK cells than the max contribution of other lineages. The bars are proportional to barcode size. Barcodes that are >1% (thus considered a biased clone in the >10x bin) are labeled in red. All other barcodes are in grey. The bar appears black due to the outline of so many small bars.

Supplemental Figure 4

(A) RhCMV in saliva and urine for the three RhCMV^{neg} RM. (B) B cells and monocytes counts in the PB post-infection. (C) Heatmap of T cells from JD76 at the 12m time point broken down by CD4 and CD8 markers. The expanded T cell clones are the dark red, enriched in the CD8+ population.
